# Early Detection of Postpartum Depressive Symptoms in Mothers and Fathers and Its Relation to Midwives’ Evaluation and Service Provision: A Community-Based Study

**DOI:** 10.3389/fped.2015.00062

**Published:** 2015-07-08

**Authors:** Jana Anding, Bernd Röhrle, Melita Grieshop, Beate Schücking, Hanna Christiansen

**Affiliations:** ^1^Department of Psychology, Child and Adolescent Psychology, Philipps-University Marburg, Marburg, Germany; ^2^EH Berlin, Berlin, Germany; ^3^Leipzig University, Leipzig, Germany

**Keywords:** postpartum emotional distress, midwives, postpartum support

## Abstract

**Background:**

Postpartum parental mental health problems pose a serious risk for child development and often remain undetected in postpartum primary care. Within the framework of the German Midwifes Prevention Study, the aim of this study was to investigate the presence of postpartum emotional distress in mothers and fathers, and the detection of distressed parents by midwives in a primary care setting. We also examined whether a temporal extension of the postpartum midwife care period is associated with greater use of midwife contacts and higher rates of referral to further professional support if needed.

**Methods:**

Mothers, fathers, and midwives filled out questionnaires at 2 weeks (*t*_1_) and 6 months (*t*_2_) postpartum. Compared to standard care in the control group (CG), midwives in an intervention group (IG) offered extended postpartum care of 6 months postpartum. Parental psychological distress was assessed using the Edinburgh postnatal depression scale (EPDS). Midwives reported on parental psychological distress as well as the number of postpartum contacts and referrals to additional social- and health-care providers.

**Results:**

Based on their ratings, midwives identified half of mothers and around one-quarter of fathers with elevated depressive symptoms according to the EPDS at *t*_1_ and *t*_2_. IG mothers used significantly more midwife contacts than CG mothers. IG mothers with high-postnatal psychological distress at *t*_2_ used significantly more contacts than mothers with lower levels of distress. IG mothers with high-psychological distress at *t*_2_ were referred to additional support services more often than mothers with lower levels of distress.

## Introduction

The birth of a child is a joyful event and a gain in resources for most families (1–3), but the transition to parenthood also requires major adaptations and involves a set of challenges and demands that can result in parental emotional distress. A significant number of mothers and fathers experience mental health problems during the postpartum period. The mean prevalence rate of postpartum depression (PPD) in women is generally between 10 and 15% (4–6). In a recent large scale study with 10,000 postpartum American women, 14% of new mothers tested positive for depressive symptoms using the Edinburgh postnatal depression scale (EPDS) (7). In a German community sample, Reck et al. (8) reported a somewhat lower prevalence rate of 8.7% for elevated depressive symptoms measured with the EPDS within the first 3 months postpartum. Although research has strongly focused on postnatal mental health in mothers, the postpartum period is also challenging for fathers (9). There is a growing body of research showing that men can also be affected by postnatal mental health problems, including depressive symptoms (10–14). A meta-analysis on paternal pre- and postnatal depression found an overall prevalence rate of 10.4%, with substantially higher rates postpartum than during pregnancy (15). Gawlik et al. (16) recently reported a prevalence of 7.8% for elevated depressive symptoms measured with the EPDS in fathers 4–6 weeks postpartum in a German community sample.

Postpartum depressive symptoms have been associated with a range of socio-demographic and psychosocial risks. Maternal peri- and postnatal risk factors for depressive mood include prenatal depression and anxiety, low self-esteem, parenting stress, life stress, lack of social support, marital relationship problems, lifetime history of depression, difficult infant temperament, maternity blues, marital status, low socioeconomic status, and unwanted/unplanned pregnancy (17–19). For fathers, Wee et al. (20) found that having a partner with depression, low marital satisfaction, and low social support were the most common correlates of postnatal depressive symptoms reported in the literature.

There is ample evidence that postpartum parental mental health problems present a serious risk to child development (21–23). While the association between maternal PDD and infant outcomes has been extensively studied, more recent studies also investigated the negative effects of different forms of maternal and paternal psychological distress, including parenting stress and anxiety symptoms (24, 25).

Long-lasting negative effects of postnatal parental distress have been attributed to impaired early parent–child interactions and parenting behavior (26–28). During infancy, maternal and paternal emotional distress is associated with impaired parent–child bonding (29), impaired cognitive and motor development (30), and infant regulatory problems [e.g., excessive crying, sleeping, and feeding problems; (31–33)], which in turn adversely affect the child’s later development (34, 35). Although rare, parental mental health problems are also known to be associated with a heightened risk for child maltreatment (36, 37). Beyond infancy, parental postpartum emotional distress is longitudinally linked to impaired cognitive and social–emotional development in preschool (14, 38) and school-aged (39) children. Parental perinatal psychological distress was even found to be a significant risk factor for adult offspring psychopathology (40).

Because of the significant long-term effects of impaired postnatal parental mental health and early parenting, a multitude of early preventive interventions have been developed that use different approaches to support parents and foster healthy living conditions for children. Existing programs mostly target at-risk families and many are home based to serve families that may otherwise be difficult to engage in supportive services (41). There is a meta-analytic evidence for the effectiveness of postnatal support programs provided to selected high-risk samples (i.e., low-income families, teenage mothers) that enhance positive maternal behavior, which in turn promotes positive child outcomes (41–43). Home visitation programs were found to be more effective when there was frequent contact (44). While the results on selective interventions to support high-risk families are promising, no evidence was found for universal beneficial effects of early preventive interventions in unselected low-risk samples (45).

In light of this research, it seems reasonable to include universal screening procedures in primary care services to identify families in need of more specialized interventions (46). This is highlighted by the fact that, in spite of the increasing awareness of parental postnatal mental health issues, maternal psychological distress frequently remains undetected, even though childbearing women are in regular, routine contact with health professionals (47–50). Identification of distressed or at-risk parents can therefore be considered as a substantial issue in postnatal care settings, as it provides a necessary pre-condition for referral and intervention (51). The systematic use of self-report screening questionnaires, such as the EPDS, has been shown to increase the detection of women at-risk for postnatal depression compared with routine clinical assessment (52, 53).

Midwives play a key role in the German pre-, peri-, and postnatal primary care system, as they have low-threshold and non-stigmatizing access to their clients and their work is highly accepted in most families (54). Especially in the early postpartum period, parents are open to changes in behavior and attitudes, and are willing to accept offers of support (55). Midwives are therefore in a key position to support new families and arrange referrals to specialist services if needed (56).

In standard care after delivery, the German compulsory health insurance service catalog covers up to 36 midwife contacts within the first 8 weeks postpartum. The first 20 contacts can be used within the first 10 days, and the remaining 16 can be distributed until the end of the eighth week of the postpartum period. Beyond the end of the eighth week, the service catalog covers up to four additional midwife contacts for breast-feeding problems. If more midwife contacts are needed, the mother needs a doctor’s certificate. No reliable nationwide data are available on how many families in Germany actually use the maximum number of midwife contacts covered by health insurance, but presumably only a minority actually receive full care.

Within the framework of the German Midwife Prevention Study [Hebammenpräventionsstudie; HPS; see Ref. (57)], the postpartum care period was extended from 8 weeks to 6 months to promote greater use of regular postnatal midwife support. The assumption was that women would receive more regular midwife visits they are entitled to if they could use this service over a longer time period. Despite the rather sobering international findings with respect to the effectiveness of universal early interventions (45), the expansion of the postnatal care period within HPS was not linked to the presence of specific risk factors in families. This approach was chosen because of the possibility that more midwife contacts would foster detection of risks and burdens in families in a non-stigmatizing way, and thus, lead to higher referral rates to specialist service providers if necessary. In this way, a prolonged care period could have a preventive effect.

The aims of the present study are to investigate

(1) the presence of elevated postnatal depressive symptoms in mothers and fathers shortly after birth and at 6 months postpartum, and midwife detection of mothers and fathers with elevated levels of psychological distress;
(2) whether extending the postnatal care period promotes greater use of postpartum midwife contacts, and whether parents with high-postnatal distress use more midwife contacts;
(3) the provision of information and referrals to other professional health- and social-care providers by midwives.

## Materials and Methods

### Study design

A quasi-experimental design was used, since random assignment of families to experimental conditions was not possible due to administrative concerns. At first, participating midwives in comparable model and control regions in Bavaria and Rhineland-Palatinate (Germany) were assigned to either the intervention group (IG) offering prolonged postpartum midwifery care or to the control group (CG) offering standard care, according to the region that they were working. The IG postpartum care period ranged from birth to the end of the sixth month postpartum period. CG postpartum care also began with birth, but ended at the end of the eighth week, reflecting the current standard of care in Germany. Groups therefore only differed with respect to the extended time interval of the IG, in which women could use their standard midwife contacts, which are normally restricted till the end of the eighth week postpartum. For comparative measurement, an additional contact at the end of the sixth month was made in the CG. Since participation of midwifes in the CG was low, midwifes were later assigned in either to the IG or CG according to study registration. While participating in the study, mothers and fathers as well as their midwives filled out questionnaires at two time points. The first-time point (*t*_1_) was within the first 2 weeks after delivery and the second time point (*t*_2_) was approximately 6 months later. Data collection was conducted from November 2010 to October 2012. Mothers and fathers were recruited for study participation by their midwives, who participated in the HPS and who provided oral as well as written information about study participation. After providing written informed consent, questionnaires were handed over to the parents by their midwives, who returned them by mail to the study administration at the Philipps-University of Marburg. Midwives filled out online questionnaires. Since parents handed over the completed questionnaires to their midwives in a closed envelope, midwifes were blind to parent data. The study protocol was approved by the ethics committee of the Psychology Faculty of Philipps-University of Marburg, Germany.

### Participants

#### Inclusion Criteria

According to the inclusion criteria for study participation, participating midwives approached mothers who had delivered live singleton infants if they were at least 18 years of age (full legal age) and insured by the Allgemeine Ortskrankenkassen (AOK).

#### Sample

Out of the 240 midwifes who originally registered for study participation, only 104 midwives (IG = 73; CG = 31) finally participated in the study. Reasons for study drop out were investigated in a sub-sample of 73 (IG = 54; CG = 19) midwifes via short telephone interviews. The main reason for study drop out according to the midwives was that not enough of their clients were insured with the AOK, which was an inclusion criterion for study participation. Another reason frequently mentioned was that they did not want to burden families with the questionnaire battery shortly after birth. At *t*_1_, questionnaires from 320 mothers and 285 fathers were sent back to the study administrators. Because recruitment was conducted by midwives, the non-responder rate is not available, since they did not assess this. Midwives themselves filled out online questionnaires for 323 families. For the purpose of the current analyses, data sets were only included if the *t*_1_ questionnaire from the mother was available. This led to the exclusion of 38 midwife questionnaires and 9 father questionnaires. Three mothers were excluded because they were younger than 18 years of age, leading to a final sample size of 317 mothers, 276 fathers, and 285 corresponding midwife questionnaires. As can be seen in Table 1, sample size was unequally distributed between groups. At *t*_2_, questionnaires from 225 mothers, 194 fathers, and 211 midwives were available. For the purpose of *t*_1_–*t*_2_ comparisons, questionnaires were only included in the data analyses if the corresponding questionnaire from both assessments were available, leading to a final sample of 216 mothers, 185 fathers, and 198 midwives. Compared to the *t*_1_ sample, the drop-out rate was 31.86% for mothers (IG: 27.3%, CG: 48.53%), 32.97% for fathers (IG: 27.62%, CG: 50%), and 30.53% for midwives (IG: 24.12%, CG: 56.14%).

**Table 1:** **Study sample (number of available questionnaires from parents and midwives) at *t*_1_ and *t*_2_**.

	*t*_1_			*t*_2_		
	Total sample	IG	CG	Total sample	IG	CG
Mothers	317	249	68	216	181	35
Fathers	276	210	66	185	152	33
Midwives	285	228	57	198^a^	173	25

*t_1_ = within 2 weeks postpartum; *t*_2_ = 6 months postpartum; IG, intervention group; CG, control group*.

*^a^For 28 *t*_2_ midwife questionnaires, the corresponding *t*_2_ mother questionnaire was not available*.

### Measures

Mothers, fathers, and midwives completed a battery of questionnaires at *t*_1_ and *t*_2_. Only those measures relevant to the purpose of this study are described here; for details of the study, see Anding et al. (57).

#### Socio-Demographic Information

The *t*_1_ questionnaires for mothers and fathers covered demographic questions regarding age, highest level of school education (0 = no school graduation, 4 = high school graduation), country of birth (as an indicator of immigration status), number of inhabitants of residence (1 = <500 to 8 = more than 1,000,000) and mothers were asked about their number of children.

A basic questionnaire for midwifes covered demographic questions regarding age, immigration background, and work experience (years they have been actively working in their profession) and was filled out by midwifes at the time of study registration.

#### Measures of Parental Emotional Distress

##### Edinburgh postnatal depression scale

The German version of the EPDS (58, 59) was used to assess depressive symptoms in mothers and fathers at *t*_1_ and *t*_2_. The EPDS is an internationally established and widely used 10-item self-report screening questionnaire to identify PPD. However, the EPDS is not a diagnostic instrument and can therefore not be used for clinical diagnosis [see, e.g., Ref. (60)]. Originally designed to screen for postnatal depression in new mothers, the EPDS has also been validated for the use in fathers (13, 61). In this study, the recommended cut-off scores of ≥13 for mothers (58) and ≥11 for fathers (61) were used to detect cases of probable major depression in parents.

##### Rating of parental emotional distress by midwives

At *t*_1_ and *t*_2_, midwives were asked if the mother/father was experiencing a high level of psychological distress (binary item). The question included no further definition of the term “psychological distress,” but relied on the midwives’ expertise. Midwives were blind to parental EPDS scores.

#### Number of Postpartum Midwife Contacts

At 6 months postpartum, midwives reported on all postpartum contacts with their clients, including the date of contact. Three different time windows were considered in the analyses: the first-time window (time 1) started at birth and covered the first 10 days postpartum, the second time window (time 2) covered the period from the 11th day to the end of the eighth week postpartum (the end of the regular postnatal care period), and the last time window (time 3) covered the remaining time from the beginning of the ninth week until the end of the sixth month postpartum. Midwife contacts were defined as the total number of contacts reported by the midwives and no further differentiation was made with respect to the type of contact.

#### Information Brokering and Referrals by Midwives, and Parental Use of Professional Support

At *t*_2_, midwives were asked if they had provided information about further professional support services (e.g., medical services or youth welfare services) to families, and if they had referred the family to any kind of professional support service (binary items). Independently of their referrals, midwives were also asked whether the family was currently using further professional support (binary item).

At *t*_2_, mothers and fathers were asked whether they were currently using professional mental health services (binary item).

### Statistical analyses

Preliminary analyses were performed to check for baseline group differences in socio-demographic measures and parental EPDS scores. To analyze group differences in metric variables, separate multivariate ANOVAs were performed for mothers’ and fathers’ socio-demographic characteristics (age, education, and number of children for mothers) and EPDS scores. Group differences in the distribution of categorical variables were evaluated using Pearson’s χ^2^ tests. The same analyses were performed to compare mothers who dropped out of the survey and mothers for whom data from both time points were available. Baseline group differences between IG and CG were then included as covariates in the subsequent analyses of variance.

The number of midwife contacts was compared between groups using a repeated-measures ANCOVA to analyze the temporal distribution of contacts across the three postpartum time intervals (with time as a within-subject factor and group as a between-subject factor). To investigate midwives’ identification of highly distressed parents, agreement between parental self-report of postnatal distress (EPDS) and midwives’ ratings of psychological distress was calculated. Data about midwife information brokering and referrals in the IG and CG were compared using Pearson’s χ^2^ tests. Partial eta squared ($\eta_{\text{p}}^{2}$) was used as an indicator of effect size in all analyses of variance. A significance level of *p* = 0.05 (two-tailed) was used for all analyses. All analyses were performed with SPSS 20 (SPSS Inc., Chicago, IL, USA).

## Results

### Preliminary analyses

#### Baseline Sample Characteristics

Descriptive statistics are shown in Table 2. There was a small but significant difference in socio-demographic characteristics between IG and CG mothers [*F*(3,313) = 3.562, *p* = 0.015; Wilks’ Λ = 0.967; $\eta_{\text{p}}^{2}=0.0\text{33}$], such that CG mothers were significantly older [*F*(1,315) = 7.227, *p* = 0.008; $\eta_{\text{p}}^{2}=0.0\text{22}$] and had a higher level of education [*F*(1,315) = 4.348, *p* = 0.038; $\eta_{\text{p}}^{2}=0.0\text{14}$]. The number of children did not differ [*F*(1,315) = 1.047, *p* = 0.307; $\eta_{\text{p}}^{2}=0.00\text{3}$]. The same pattern was observed for fathers [*F*(2,273) = 5.685, *p* = 0.004; Wilks’ Λ = 0.960; $\eta_{\text{p}}^{2}=0.0\text{4}0$] with respect to age [*F*(1,274) = 7.296, *p* = 0.007; $\eta_{\text{p}}^{2}=0.0\text{26}$] and education level [*F*(1,274) = 6.162, *p* = 0.014; $\eta_{\text{p}}^{2}=0.0\text{22}$]. ANOVAs revealed no group differences in EPDS scores for mothers [*F*(1,315) = 0.278, *p* = 0.592; $\eta_{\text{p}}^{2}=0.00\text{1}$] or fathers [*F*(1,274) = 0.377, *p* = 0.540; $\eta_{\text{p}}^{2}=0.00\text{1}$]. One hundred and seventy-five (55.2%) mothers in the sample were primipara, and 174 (54.9%) of the newborn babies were male. The proportion of first-time mothers [χ^2^(1) = 2.323, *p* = 0.133] and the distribution of child gender [χ^2^(1) = 0.409, *p* = 0.583] did not differ between groups. About 18% of mothers and 15% of fathers were born in another country, with no differences between groups [mothers: χ^2^(1) = 1.405, *p* = 0.288; fathers: χ^2^ = 2.915, *p* = 0.092]. Because of the baseline group differences, maternal and paternal age and education level were included as covariates in the analyses of variance.

**Table 2:** **Descriptive statistics of parental socio-demographic characteristics and EPDS scores at *t*_1_ and *t*_2_**.

		Total (*N* = 317)		IG (*N* = 249)		CG (*N* = 68)	
		*M*	SD	*M*	SD	*M*	SD
**Mothers**	Age	28.83	5.22	28.42	5.16	30.32	5.18
	Education	2.89	0.83	2.84	0.84	3.08	0.77
	Number of children	1.57	0.78	1.54	0.80	1.65	0.72
	EPDS *t*_1_	7.22	5.06	7.14	5.04	7.52	5.14
	EPDS *t*_2_^a^	5.19	4.71	5.03	4.54	6.07	5.49
	Number of inhabitants^c^	3.76	1.82	3.74	1.79	3.85	1.95

		**Total (*N* = 267)**		**IG (*N* = 210)**		**CG (*N* = 66)**	

**Fathers**	Age	32.18	6.21	31.62	6.28	33.96	5.66
	Education	2.75	0.88	2.68	0.87	2.99	0.87
	EPDS *t*_1_	4.27	3.57	4.20	3.41	4.51	4.06
	EPDS *t*_2_^b^	3.76	3.67	3.98	3.65	2.76	3.67

*t_1_ = within 2 weeks postpartum; *t*_2_ = 6 months postpartum; IG, intervention group; CG, control group; total, total sample*.

*^a^Total *N* = 216; IG *N* = 181; CG *N* = 35*.

*^b^Total *N* = 185; IG *N* = 152; CG *N* = 33*.

*^c^Number of inhabitants (1 = <500, 2 = 500–1000, 3 = 1000–2000, 4 = 2000–5000, 5 = 5000–20,000, 6 = 20,000–100,000, 7 = 100,000–1,000,000, 8 = more than 1,000,000)*.

#### Socio-Demographic Characteristics and Qualification of the Midwives

The mean age of the midwives was 40.33 (SD = 9.06) years and their mean work experience (years they have been working in their profession) was 15.68 (SD = 8.73) years, with no significant differences between IG and CG [age: *F*(1,84) = 0.432, *p* = 0.513; $\eta_{\text{p}}^{2}=0.00\text{5}$; work experience: *F*(1,75) = 0.009, *p* = 0.926; $\eta_{\text{p}}^{2}<0.00\text{1}$]. A total of 23.1% of the midwives had an immigration background, again with no difference between groups [χ^2^(1) = 1.730, *p* = 0.231].

#### Drop-Out Analyses

There were no significant differences in age, education, and number of children between mothers who dropped out of the survey (*N* = 101) and mothers who participated at both time points (*N* = 216) [*F*(3,313) = 1.620, *p* = 0.060; Wilks’ Λ = 0.982; $\eta_{\text{p}}^{2}=0.0\text{14}$]. There was a significant difference in EDPS scores [*F*(1,315) = 8.629, *p* = 0.004; $\eta_{\text{p}}^{2}=0.0\text{27}$], such that mothers who dropped out had higher EPDS scores (*M* = 8.43, SD = 5.34 versus *M* = 6.66, SD = 4.83). There were no differences in the proportion of primipara [χ^2^(1) = 2.683, *p* = 0.115], immigration background [χ^2^(1) = 0.653, *p* = 0.255], or child gender [χ^2^(1) = 2.527, *p* = 0.117].

### Parental psychological distress

#### Identification of Highly Distressed Parents at *t*_1_

Edinburgh postnatal depression scale cut-off scores of ≥13 (mothers) and ≥11 (fathers) were used to identify parents with high-psychological distress. There was no difference in the percentage of highly strained mothers and fathers between groups. Frequencies and inferential statistics are shown in Table 3.

**Table 3:** **Number and proportion of mothers and fathers scoring above and below EPDS cut-offs at *t*_1_ and *t*_2_ and inferential statistics of between group comparisons**.

		*t*_1_					*t*_2_				
		Total	IG	CG	χ^2^	*p*	Total	IG	CG	χ^2^	*p*
		
		*N* (%)	*N* (%)	*N* (%)			*N* (%)	*N* (%)	*N* (%)		
**Mothers**	EPDS ≥ 13	51 (16.1)	41 (16.5)	10 (14.7)	0.123	0.853	20 (9.3)	16 (8.8)	4 (11.4)	0.234	0.629
	EPDS ≤ 12	266 (83.9)	208 (83.5)	58 (85.3)			196 (90.7)	165 (91.2)	31 (88.6)		
**Fathers**	EPDS ≥ 11	15 (5.4)	11 (5.2)	4 (6.1)	0.066	0.761	11 (5.9)	9 (5.9)	2 (6.1)	0.001	0.975
	EPDS ≤ 10	261 (94.6)	199 (94.8)	62 (93.9)			174 (94.1)	143 (94.1)	31 (93.9)		

*N, number of mothers/fathers below or above EPDS cut-offs; IG, intervention group; CG, control group; total, total sample, *t*_1_ = within 2 weeks postpartum, *t*_2_ = 6 months postpartum*.

Midwives rated 25% of mothers and 10% of fathers as having high-psychological distress (see Table 4). While there was no difference in midwife ratings between groups for mothers, there was a difference in ratings for fathers [χ^2^(1) = 5.35, *p* = 0.022], because only one CG father was rated as heavily distressed.

**Table 4:** **Numbers and percentages of psychological distressed parents at *t*_1_ and *t*_2_ according to midwifes’ judgments and inferential statistics**.

		*t*_1_					*t*_2_				

		Total	IG	CG	χ^2^	*p*	Total	IG	CG	χ^2^	*p*
**Mothers^a^**	*N* (%)	72 (25.3)	61 (26.8)	11 (19.3)	1.34	0.307	40 (20.2)	35 (20.2)	5 (20)	0.001	0.979
**Fathers^b^**	*N* (%)	27 (9.9)	26 (12.0)	1 (1,8)	5.35	0.022	17 (9.1)	14 (8.6)	3 (12.5)	0.387	0.534

*^a^Midwives judgments of mothers’ psychological distress (*t*_1_: *N* = 285; *t*_2_: *N* = 198)*.

*^b^Midwives’ judgments of fathers’ psychological distress (*t*_1_: *N* = 273; *t*_2_: *N* = 187)*.

*Total, total sample; IG, intervention group; CG, control group; *t*_1_ = within 2 weeks postpartum; *t*_2_ = 6 months postpartum*.

Agreement between self-report of parental distress (EPDS) and midwife ratings was inspected when both parental and midwife ratings were available (Table 5). Midwives identified 47.83% (22/46) of mothers and 21.43% (3/14) of fathers with high EPDS scores. The rates within groups were 45.94% (17/37) and 27.27% (3/11) for IG mothers and fathers, respectively, and 55.56% (5/9) and 0% (0/3) for CG mothers and fathers, respectively.

**Table 5:** **Number and percentages of highly distressed parents (EPDS > cut-off) identified as highly distressed by midwife ratings at *t*_1_ and *t*_2_**.

	*t*_1_			*t*_2_		
	Total	IG	CG	Total	IG	CG
Mothers with EPDS ≥ 13 identified as distressed^a^	22/46 (47.83%)	17/37 (45.94%)	5/9 (55.56%)	9/18 (50.00%)	7/15 (46.67%)	2/3 (66.67%)
Fathers with EPDS ≥ 11 identified as distressed^a^	3/14 (21.43%)	3/11 (27.27%)	0/3 (–)	2/8 (25.00%)	2/8 (25%)	–

*^a^Midwife judgment on whether the mother/father is experiencing high levels of psychological distress (yes/no)*.

*Total, total sample; IG, intervention group; CG, control group; *t*_1_ = within 2 weeks postpartum; *t*_2_ = 6 months postpartum*.

#### Identification of Highly Distressed Parents at *t*_2_

Descriptive statistics of parental EPDS scores at *t*_2_ are shown in Table 2. At 6 months postpartum, 10% of mothers and 6% of fathers had EPDS scores above the cut-off. Again, there was no difference in the percentage of highly strained parents between groups (see Table 3).

Midwives identified 20% of mothers and 9% of fathers as highly distressed at 6 months postpartum. There was no difference between groups regarding the proportion of emotionally distressed mothers and fathers.

Agreement between parental distress according to EPDS scores and midwife ratings was inspected when both parental and corresponding midwife ratings were available (Table 5). At 6 months postpartum, midwives identified 50% (9/18) of mothers and 25% (2/8) of fathers with high EPDS scores. In the IG, 46.67% (7/15) of mothers and 25% (2/8) of fathers with high EPDS scores were judged as heavily distressed by midwives. In the CG, 66.67% (2/3) of mothers with high EPDS scores were also judged as heavily distressed by midwives. No CG fathers had high EPDS scores or were judged as heavily distressed by midwives.

### Number of midwife contacts

Data on postpartum midwife contact were not available in 21 cases, leading to a sample of *N* = 177 data sets for statistical analyses. Descriptive statistics for the number of midwife contacts are presented in Table 6. A repeated measure ANCOVA [with time as a within-subject factor (three levels: time 1, time 2, time 3), group as a between-subject factor (IG, CG), and age and education as covariates] was performed. Mauchly’s test indicated that the assumption of sphericity was violated [χ^2^(2) = 31.486, *p* < 0.001], therefore, degrees of freedom were corrected using Greenhouse–Geisser estimates of sphericity (ε = 0.857). There was a significant main effect of group [*F*(1,173) = 9.92, *p* = 0.002, $\eta_{\text{p}}^{2}=0.0\text{54}$], indicating that IG mothers used more midwife contacts than CG mothers, and a significant interaction between time and group, indicating differences in the temporal distribution of contacts between groups [*F*(1.713,296.415) = 10.373, *p* < 0.001, $\eta_{\text{p}}^{2}=0.0\text{57}$]. The main effect of time was not significant [*F*(1.713,296.415) = 1.757, *p* = 0.174, $\eta_{\text{p}}^{2}=0.0\text{1}0$]. Further univariate ANCOVAs revealed a higher use of contacts in the IG versus CG group between day 11 and the end of week 8 postpartum [*F*(1,173) = 4.062, *p* = 0.045, $\eta_{\text{p}}^{2}=0.0\text{23}$], and between 9 weeks and 6 months postpartum [*F*(1,173) = 16.056, *p* < 0.001, $\eta_{\text{p}}^{2}=0.0\text{85}$], whereas there was no significant difference between groups within the first 10 days postpartum [*F*(1,173) = 0.001, *p* = 0.971, $\eta_{\text{p}}^{2}=0.000$].

**Table 6:** **Descriptive statistics for the number and temporal distribution of postpartum midwife contacts**.

	Total (*N* = 177)		IG (*N* = 151)		CG (*N* = 26)	
	*M*	SD	*M*	SD	*M*	SD
Total number of contacts	20.95	9.68	21.90	9.58	15.46	8.50
Time 1	4.49	2.82	4.49	2.90	4.5	2.37
Time 2	8.52	3.96	8.76	3.98	7.12	3.60
Time 3	7.952	5.67	8.65	5.47	3.92	5.19

*Total, total sample; IG, intervention group; CG, control group; total number of contacts, number of midwife contacts between day of birth and the end of the sixth month postpartum; Time 1, number of contacts within the first 10 days postpartum; Time 2, number of contacts between the 11th day and the end of the eighth week postpartum; Time 3, number of contacts between the beginning of the ninth week and the end of the sixth month postpartum*.

Next, contact frequency was compared between mothers with high levels of postnatal depressive symptoms (EPDS ≥ 13) within the first 2 weeks postpartum (*t*_1_) and mothers who did not show elevated symptoms at *t*_1_. Because data on midwife contacts were only available for three CG mothers with elevated *t*_1_ EPDS scores, no comparison between mothers with elevated *t*_1_ EPDS scores in the IG versus CG was conducted, and only IG data were analyzed (*N* = 142). Contact data were available for 22 IG mothers who had high *t*_1_ EPDS scores. The average number of contacts between birth and the sixth month postpartum was 21.59 (SD = 9.95) for mothers with *t*_1_ EPDS ≤ 12 (*N* = 129), and 23.68 (SD = 6.94) for mothers with *t*_1_ EPDS ≥ 13. A repeated-measures ANCOVA [with time (three levels: time 1, time 2, time 3) and maternal distress at *t*_1_ (EPDS ≤ 12, EPDS ≥ 13) as within-subjects factors, and age and education as covariates] was performed. Mauchly’s test indicated that the assumption of sphericity was violated [χ^2^(2) = 24.416, *p* < 0.001], therefore, degrees of freedom were corrected using Greenhouse–Geisser estimates of sphericity (ε = 0.867). There was a significant main effect of time [*F*(1.733, 254.767) = 3.472, *p* = 0.039, $\eta_{\text{p}}^{2}=0.0\text{23}$], but no significant interaction between time and maternal distress at *t*_1_ [*F*(1.733, 254.767) = 0.656, *p* = 0.499, $\eta_{\text{p}}^{2}=0.00\text{4}$], and no significant main effect of maternal distress at *t*_1_ [*F*(1,147) = 1.010, *p* = 0.317, $\eta_{\text{p}}^{2}=0.00\text{7}$].

The same analysis was performed to compare contact frequency of mothers with *t*_2_ EPDS scores ≥13 to mothers with *t*_2_ EPDS scores ≤12. Again, because data on midwife contacts were only available for three CG mothers with elevated *t*_2_ EPDS scores, no comparison between mothers with elevated *t*_2_ EPDS scores in the IG versus CG was conducted, and only IG data were analyzed. Because maternal *t*_2_ EPDS scores and data on midwife contacts were required for this comparison, the sample was further reduced to *N* = 132. The average total number of contacts between birth and 6 months postpartum was 21.46 (SD = 8.841) for mothers with *t*_2_ EPDS ≤ 12 (*N* = 119), and 27.54 (SD = 16.16) for mothers with *t*_2_ EPDS ≥ 13 (*N* = 13).

A repeated-measures ANCOVA [with time (three levels: time 1, time 2, time 3) and maternal distress at *t*_2_ (EPDS ≤ 12, EPDS ≥ 13) as within-subject factors, and age and education as covariates] was performed. Mauchly’s test indicated that the assumption of sphericity was violated [χ^2^(2) = 18.715, *p* < 0.001], therefore, degrees of freedom were corrected using Greenhouse–Geisser estimates of sphericity (ε = 0.879). There was no significant main effect of time [*F*(1.759, 225.15) = 2.921, *p* = 0.063, $\eta_{\text{p}}^{2}=0.0\text{22}$], and no significant interaction between time and maternal distress at *t*_2_ [*F*(1.759, 225.15) = 3.038, *p* = 0.057, $\eta_{\text{p}}^{2}=0.0\text{23}$]. However, there was a significant main effect of maternal distress at *t*_2_ [*F*(1,128) = 4.447, *p* = 0.037, $\eta_{\text{p}}^{2}=0.0\text{34}$], indicating that mothers with *t*_2_ EPDS ≥ 13 used significantly more midwife contacts.

### Information brokering and referrals to professional support providers

Midwives stated that they provided information about professional support to 58% of their clients. About 10% of the families were referred to other professional health- or social-care providers by midwives. Irrespective of their referrals, midwives reported that 14% of families were using additional professional support. There were no significant associations between group and information provided, referrals to professional services, and use of professional services (see Table 7). Eleven mothers and two fathers reported that they were currently undergoing psychiatric or psychotherapeutic treatment (see Table 7). Of those mothers who reported that they were currently receiving professional mental health treatment, 63.63% (7/11) had *t*_2_ EPDS scores ≥13. Thus, 35% (7/20) of the mothers with *t*_2_ EPDS scores ≥13 were receiving mental health treatment.

**Table 7:** **Information provided, referrals, and use, and inferential statistics**.

		Total		IG		CG		χ^2^	*p*
		*N*	%	*N*	%	*N*	%
**Information^a^**	Yes	115	58.1	104	60.1	11	44.0	2.33	0.136
	No	83	41.9	69	39.9	14	56.0		
**Referrals^b^**	Yes	19	9.6	16	9.2	3	12.0	0.191	0.662
	No	179	90.4	157	90.8	22	88.0		
**Use^c^**	Yes	28	14.1	26	15.0	2	8.0	0.889	0.540
	No	170	85.9	147	85.0	23	92.0		
**Treatment^d^**
**Mothers**	Yes	11	5.1	8	4.5	2	8.0	1.010	0.394
	No	203	94.9	171	95.5	23	92.0		
**Fathers**	Yes	2	1.1	2	1.3	0	–	–	–
	No	182	98.9	149	98.7	33	100		

*Total, total sample; IG, intervention group; CG, control group*.

*^a^Midwife has informed family about professional support (yes/no)*.

*^b^Midwife has referred family to professional support providers (yes/no)*.

*^c^Family actually uses professional support*.

*^d^Mother/father currently receives professional mental health treatment*.

Due to the small sub-sample of CG mothers with *t*_2_ EPDS ≥ 13 (*N* = 4) and the small total number of referrals and uses in the CG, further analyses of associations between maternal distress, referrals, and use rates were only conducted for the IG (see Table 8). Information provided, referrals, and use rates were significantly associated with maternal distress (*t*_2_ EPDS ≥ 13) in the IG, indicating that mothers with high distress were more frequently informed about additional support, were more frequently referred to corresponding services by midwives, and were more frequently using them, compared to mothers with lower distress.

**Table 8:** **Information provided, referrals, and use for IG mothers with EPDS ≥13 and ≤12, and inferential statistics**.

		*t*_2_ EPDS ≥ 13		*t*_2_ EPDS ≤ 12		χ^2^	*p*
		*N*	%	*N*	%
**Information^a^**	Yes	13	86.7	75	55.6	5.389	0.020
	No	2	13.3	60	44.4		
**Referrals^b^**	Yes	6	40.0	6	4.4	23.188	<0.001
	No	9	60.0	129	95.6		
**Use^c^**	Yes	8	53.3	13	9.6	21.416	<0.001
	No	7	46.7	122	90.4		
**Treatment^d^**	Yes	5	31.3	3	1.8	29.515	<0.001
	No	11	68.8	160	98.2		

*^a^Midwife has informed family about professional support (yes/no)*.

*^b^Midwife has referred family to professional support providers (yes/no)*.

*^c^Family actually uses professional support*.

*^d^Mother/father currently receives professional mental health treatment*.

## Discussion

A significant number of parents reported high levels of psychological distress shortly after birth and at 6 months postpartum. The rates of elevated depressive symptoms of 16 and 9% for mothers at *t*_1_ and *t*_2_, respectively, and around 6% at both time points for fathers in the present study are consistent with other research on the prevalence of postnatal parental psychological distress in Germany (8, 16). Midwives’ ratings suggested higher prevalence rates of parental psychological distress than parental self-report. Within 2 weeks postpartum, midwives rated 25% of mothers and 10% of fathers as highly distressed. At 6 months postpartum, midwives rated 20 and 9% of mothers and fathers, respectively, as highly distressed. Even though these rates are quite high, this result is consistent with other research. For example, in a study with German, Swiss, and Austrian midwives, midwives retrospectively estimated that about one-third of the women that they were caring for were experiencing high levels of emotional distress (56).

Midwives identified 48% (*t*_1_) and 50% (*t*_2_) of women with high EPDS scores. This indicates that 50% of the mothers experiencing significant postnatal depressive symptoms remained undetected by midwives. In the present study, midwives were blind to parents’ EPDS scores and judgments relied on their expertise and work experience. This is consistent with other research on the detection of postnatal depressive symptoms in routine postnatal care when no systematic identification strategy is in use. For example, in a study by Morris-Rush et al. (50), primary care providers who were blind to mothers’ EPDS scores identified 14/27 (51.9%) of mothers with elevated symptoms. In other studies, detection rates were even lower, and ranged around 30% (48). Since early detection of women with increased emotional distress provides an opportunity for referral and early intervention, and may therefore prevent serious long-term consequences, active identification strategies including routine screening in primary care seem reasonable (51, 62). Positive effects of training on postnatal depression screening and implementation of standardized tools in primary care on the detection of highly distressed mothers have already been reported in the literature (52, 62).

To our knowledge, the present study is the first to investigate detection of postnatal psychological distress in fathers in a primary postnatal care setting. Midwife detection rates for fathers with high levels of postnatal depression symptoms were found to be substantially lower than for mothers, with only 21% (*t*_1_) and 25% (*t*_2_) identified. Thus, the vast majority of highly strained fathers were not recognized by midwifes in routine care after birth. However, this is not surprising, as midwife care is focused on advice, support, and care for the mother and child, and fathers are involved to a lesser degree. While postpartum mental health problems in fathers and their effects on child development have only been recently recognized in the scientific literature, this is still a neglected issue in routine care. In light of the growing body of evidence that paternal postpartum mental health problems are independently associated with detrimental effects on child development, the results of the present study underscore the fact that more research is needed to address mental health issues in new fathers.

Temporal extension of the postnatal midwife care period was associated with greater use of general midwife support. On average, IG families used six additional midwife contacts compared to CG families who received standard postpartum midwife care. However, the mean number of contacts in both groups was far below the available 36 contacts. IG mothers with high psychological distress at 6 months postpartum used significantly more midwife contacts than mothers with lower levels of postnatal distress. Although this might represent a promising prevention effect, this study does not show whether higher use is due to distressed mothers making more demands on support or midwives offering more frequent contacts to women in need of additional support. Whether this higher use of midwife contacts by highly distressed mothers is associated with potential long-term benefits to mother and child well-being cannot be addressed here because no follow-up data are available.

Midwives stated that they frequently informed their clients about additional support. General referral rates did not differ between the IG and CG, but in the IG, rates of providing information and referrals to additional support providers were significantly higher for mothers with high levels of depressive symptoms compared to mothers with lower distress levels (information: 87 versus 56%, referrals: 40 versus 4%). Although a comparison with the CG was not possible due to small sample sizes, this is a promising result, underscoring the significant position and potential of midwives within the primary postnatal care system.

The results of this study show that irrespective of referrals arranged by midwives, 53% of highly distressed mothers (versus 10% of low-distress mothers) were using additional support at 6 months postpartum. However, this implies that half of the mothers with high levels of psychological distress were not receiving any support other than standard care at the time of the survey. Of mothers with elevated depressive symptoms at 6 months postpartum, 35% reported that they were undergoing psychiatric or psychotherapeutic treatment.

The study has several methodological limitations that need to be considered. The sample size of the CG was small, and the drop-out rates from *t*_1_ to *t*_2_ was high, especially in the CG. Telephone interviews with a sub-sample of 73 midwives revealed that practical reasons (i.e., lack of clients who fit in the inclusion criteria for study participation) and apprehensions to burden families with questionnaires including very personal issues shortly after birth were the main reasons for midwives to refrain from study participation. Especially within the CG, barriers for study participation can assumed to be high, since midwives did not have the opportunity to offer prolonged service to their clients. High drop-out rates within the CG might in part be attributable to the fact that the second point of data collection (*t*_2_) was outside the regular care period so that midwives might not have been in contact with their clients anymore – even though they were offered the possibility for an additional contact at 6 months postpartum. The highest drop-out rate actually occurred for the midwife questionnaires within the CG, indicating that midwives in some cases did not complete the online questionnaire although they had sent back the completed questionnaires of their clients. However, it can be noted that other studies with a universal approach and quasi-experimental design have similar participation problems [see, for example, Ref. (63)], so that this might be less a specific feature of the HPS, but a general problem of universal approach studies with a long time frame between the measuring times, the absence of direct contacts of the participants (families) with the research institutions, and a relatively comprehensive measuring instrument. Since recruitment of families for study participation was conducted by the midwives, no information is available about the rate of non-responders. Moreover, since it cannot be ruled out that midwifes selectively approached families for study participation, generalizability of results is limited due to potential sampling bias. Drop-out analyses further revealed that dropouts and responders significantly differed with respect to maternal distress, such that women who experienced high-psychological distress at *t*_1_ were more likely to drop out of the survey than mothers with lower levels of distress at *t*_1_. Although not surprising, this of course also challenges the generalizability of our results, since it affects the central research question whether especially those women showing high levels of distress can be reached and further referred in the context of a temporal extended postnatal midwifery care period.

There were methodological differences in the assessment of psychological distress between parental self-report and midwife ratings: midwives’ ratings of parental distress were only assessed with a binary item and the term “psychological distress” was not further defined. Midwives were therefore not explicitly asked to rate parental depressive symptoms and their judgments did not include severity ratings. Midwives therefore relied on their expertise in judging whether parents were suffering high levels of psychological distress against the background that their mean work experience was around 15 years. Within the framework of the HPS, midwives were blind to parents EPDS scores and were not specifically trained or instructed to screen for mental health problems in their clients. Thus, their judgments reflect their current knowledge on parental distress.

## Conclusion

A significant number of newborn children are confronted with psychologically distressed parents, which is a major risk factor for child development (21–23). Thus, effective interventions are needed to reduce this risk. About half of the women with high-psychological distress remain undetected in routine care. Although the results of the present study suggest that a significant number of families received professional support beyond standard midwife care within the postpartum period, they also indicate that more than half of mothers with high levels of emotional distress were not receiving additional support at the time of the survey. Thus, there is a major need for better identification of distressed parents so that support can be provided to enhance positive family and child development.

## Conflict of Interest Statement

The authors declare that the research was conducted in the absence of any commercial or financial relationships that could be construed as a potential conflict of interest.
